# Long-term Clinical Course of Antineutrophil Cytoplasmic Antibody-associated Vasculitis Patients off Maintenance Therapy

**DOI:** 10.7759/cureus.2372

**Published:** 2018-03-26

**Authors:** Eric J Gapud, Rebecca Manno, Philip Seo, Mohamad Hanouneh, Duvuru Geetha

**Affiliations:** 1 Medicine, The Johns Hopkins University School of Medicine; 2 Division of Rheumatology, The Johns Hopkins University School of Medicine

**Keywords:** anca vasculitis, immunosuppression, outcomes

## Abstract

Objectives

The optimal duration of maintenance immunosuppressive therapy in patients with antineutrophil cytoplasmic antibody (ANCA)-associated vasculitis (AAV) is still controversial. The aim of our study is to describe the characteristics and outcomes of patients with AAV who were able to stop maintenance agents completely while remaining on daily prednisone (< 5 mg) for at least 36 months.

Materials and methods

AAV patients treated at our center from 2000 to 2016 and who were not on maintenance agents while remaining on prednisone < 5 mg daily for at least 36 months were identified by the providers, and their records were retrospectively reviewed. Relapse was defined by the reinitiation of immunosuppressive therapy for biopsy-proven glomerulonephritis or any extra-renal organ involvement.

Results

Of the 18 patients who fulfilled the study inclusion criteria, 12 were male and 14 were Caucasian. The mean age at AAV diagnosis was 54 years. Seventeen patients had renal involvement and seven had lung involvement. Eleven patients received cyclophosphamide and eight patients received rituximab along with glucocorticoids for remission induction. Twelve patients were weaned completely off prednisone. The median duration of prednisone use was 20 months. Nine patients received maintenance therapy with azathioprine or mycophenolate mofetil. The median duration of maintenance therapy was 24 months. The mean follow-up time after stopping the maintenance agent was 64 months. During this period, three patients had disease relapse.

Conclusions

Stopping maintenance agents for > 36 months can be achieved in some patients with AAV. Prospective, randomized controlled trials are needed to confirm this finding.

## Introduction

Antineutrophil cytoplasmic antibody (ANCA)-associated vasculitides (AAV) are necrotizing small vessel systemic diseases. Recent advances in the treatment of AAV have transformed these previously universally fatal diseases into chronic relapsing diseases, although substantial morbidity and mortality still exist related to vasculitis and treatment-associated side effects [1-2]. AAV therapy involves a two-stage approach consisting of remission induction followed by remission maintenance. Relapse is common and occurs in up to 50% of patients within five years, leading to an additional accrual of disease- and treatment-related damage [3]. Due to this high relapse rate, practitioners are constantly challenged with balancing the relapse risk with the long-term side effects of pharmacotherapy.

The optimal duration for remission maintenance therapy remains uncertain. Despite the recognition that current AAV therapies are not a cure and despite the morbidity of long-term immunosuppression, discontinuation of immunosuppressive therapy has not been explored in-depth and has generally been considered largely unachievable [4]. Current guidelines recommending continuing maintenance therapy for at least 24 months after achieving disease remission are derived merely from the duration of previous maintenance trials [5-6]. In this study, we sought to characterize outcomes in a subset of AAV patients treated at our center who have been off maintenance immunosuppressive agents for at least 36 months.

## Materials and methods

Study population

Patients for this single-center retrospective study were identified by providers at our Vasculitis Center and received care any time from 2000 to 2016. Subjects who met the 2012 Chapel Hill Classification criteria for granulomatosis with polyangiitis (GPA) or microscopic polyangiitis [7] and were off maintenance immunosuppressive agents (e.g., azathioprine, mycophenolate mofetil, methotrexate) therapy for at least 36 months were included. Patients with both a new diagnosis of AAV and a relapsing disease were included. For patients with the relapsing disease, the 36 consecutive months off immunosuppression were defined relative to the date of the last known relapse. For patients who received a renal transplant, the follow-up period ended on the transplantation date. All patients were followed until their last clinic visit date, renal transplant, or death. The School of Medicine Human Subjects Research and Institutional Review Board approved our study protocol as an exempt study. Written informed consent was not obtained and was waived due to the retrospective nature of the study.

Acquisition of clinical and laboratory data

Patient demographics, clinical features, and details of induction and maintenance immunosuppressive therapy, including start and end dates, were abstracted retrospectively from the electronic medical records. ANCA testing was done by standard indirect immunofluorescence assay on ethanol-fixed neutrophils for cytoplasmic ANCA (c-ANCA) and perinuclear ANCA (p-ANCA). Antiproteinase 3 (PR3) and myeloperoxidase (MPO) testing was done by direct enzyme-linked immunosorbent assay using commercially available kits and was performed in our clinical laboratory.

Study definitions

1. The end date of therapy was the final date of administration of azathioprine, mycophenolate mofetil, or methotrexate. If rituximab was used for maintenance therapy, the last day of therapy was considered the date when CD19 cells were documented as detectable. We also considered prednisone doses of ≤ 5 mg daily as non-therapeutic and defined the date that the dose of prednisone monotherapy was tapered to ≤ 5 mg daily as the last day of immunosuppression.

2. Renal function was estimated at diagnosis and during follow-up using the four-variable modification of diet in renal disease formula for estimated glomerular filtration rate (e-GFR) [8].

3. Renal involvement was defined as biopsy-proven glomerulonephritis or clinically by a rise in serum creatinine with hematuria and proteinuria.

4. Proteinuria was defined as a urine protein to creatinine ratio > 0.2.

5. Other organ involvement, such as lung, heart, and skin, was defined by imaging and a diagnostic biopsy.

6. Relapse was defined by biopsy-proven glomerulonephritis or extra-renal organ involvement requiring reinitiation of immunosuppressive therapy and/or prednisone > 5 mg daily.

Statistical analyses

All descriptive data are reported as median with range or mean with standard deviation (SD).

## Results

Eighteen patients fulfilled our study criteria. Baseline characteristics are shown in Table 1. The mean age at AAV diagnosis was 54 years (SD, 23 years). Fourteen patients were Caucasian. Male patients comprised 67% of the cohort. The majority of patients were MPO-ANCA positive, and seven patients had relapsing disease. Seventeen patients had renal involvement, and seven had lung involvement. The mean e-GFR at entry was 46 mL/min/m2 (SD, 39 mL/min/m2), and the mean e-GFR at one year off maintenance immunosuppressive agent was 52 mL/min/m2 (SD, 25 mL/min/m2). The mean follow-up time was 121 months (SD, 60 months).

**Table 1:** Baseline characteristics of AAV patients off maintenance immunosuppressive agents AAV, ANCA-associated vasculitis; ANCA, antineutrophil cytoplasmic antibody; F, female; M, male; AA, African American; CA, Caucasian; OT, other; MPO, myeloperoxidase; PR3, anti-proteinase; NEG, negative; S, sinus; J, joints; L, lungs; K, kidneys; SK, skin; P, peripheral nerves; PID, patient identification

PID	Age at AAV Diagnosis	Gender	Race	ANCA Type	Relapsing Disease	Organs Involved	GFR at Diagnosis (mL/min/m^2^)	Follow-Up Time (Months)
1	66	F	AA	MPO	NO	S, P, J, K	50	58
2	45	M	CA	MPO	YES	K, L	26	74
3	30	M	CA	PR3	NO	S, E, L	114	168
4	15	M	CA	MPO	YES	K, L	152	132
5	67	M	CA	MPO	NO	K, L, S, E	9	136
6	83	F	CA	MPO	NO	SK, K	36	62
7	36	M	CA	NEG	YES	K, L	43	241
8	43	M	OT	PR3	YES	J, L, S, K	41	151
9	18	F	CA	MPO	NO	K	37	67
10	52	M	CA	PR3	NO	K, SK	111	148
11	84	M	AA	MPO	NO	K	14	91
12	69	M	CA	MPO	NO	K	33	85
13	76	M	CA	PR3	NO	S, J, L, K	21	70
14	67	M	CA	MPO	NO	K	22	91
15	55	F	CA	PR3	YES	K, L, J	32	228
16	21	M	CA	PR3	YES	K, J	50	200
17	77	F	OT	MPO	NO	K	15	142
18	70	F	CA	MPO	YES	K, J	19	38

Details of the immunosuppressive therapy these patients received are presented in Table 2. The induction immunosuppression regimen for all patients included glucocorticoids. Ten patients received cyclophosphamide. The cyclophosphamide was administered to four of 11 patients intravenously, and the other seven who received cyclophosphamide were given an oral formulation. The median duration of cyclophosphamide use was six months (range, one to 12 months). Seven patients received rituximab as induction therapy. A single patient with relapsing disease received a four-week course of oral cyclophosphamide and was switched to rituximab for remission induction. Prednisone was weaned completely in 12 patients with the median duration of prednisone use among these 12 patients being 20 months (range, six to 42 months). Among the rituximab-treated patients, all four patients who were tested for B cells had evidence of B cell repopulation at the last follow-up evaluation.

**Table 2:** Details of induction and maintenance immunosuppression RTX, rituximab; CYC, cyclophosphamide; PRED, prednisone; MMF, mycophenolate mofetil; AZA, azathioprine; PID, patient identification

PID	Induction agents	Maintenance agents	Prednisone dose during maintenance/ mg	Duration of steroids use in months	Duration of other maintenance therapy in months
1	Glucocorticoids, RTX	PRED	5 mg	Ongoing	---
2	Glucocorticoids, CYC	PRED	5 mg	13	---
3	Glucocorticoids, CYC	None	---	12	---
4	Glucocorticoids, CYC	MMF, PRED	5 mg	20	45
5	Glucocorticoids, CYC	AZA, PRED	5 mg	18	6
6	Glucocorticoids, RTX	PRED	5 mg	Ongoing	---
7	Glucocorticoids, CYC, RTX	None	---	10	---
8	Glucocorticoids, RTX	AZA	---	6	69
9	Glucocorticoids, CYC	AZA, PRED	5 mg	18	29
10	Glucocorticoids, CYC	AZA, PRED	5 mg	6	86
11	Glucocorticoids, CYC	AZA, PRED	2.5 mg	Ongoing	24
12	Glucocorticoids, RTX	PRED	5 mg	42	---
13	Glucocorticoids, CYC	AZA, MMF, PRED	5 mg	Ongoing	3
14	Glucocorticoids, CYC	AZA, PRED	2.5 mg	41	23
15	Glucocorticoids, RTX	PRED	4 mg	Ongoing	---
16	Glucocorticoids, RTX	PRED	5 mg	13	---
17	Glucocorticoids, CYC	AZA, PRED	5 mg	42	12
18	Glucocorticoids, RTX	PRED	5 mg	Ongoing	---

Nine patients received maintenance therapy. The median duration of maintenance therapy for these 10 patients was 24 months (range: six to 86 months). Of the nine patients who did not receive maintenance therapy, seven were induced with rituximab and two received cyclophosphamide. The seven rituximab-induced patients were not given maintenance immunosuppression, and the two cyclophosphamide-treated patients were intolerant of azathioprine and were given an extended course with cyclophosphamide instead. Three patients did not receive prednisone during the maintenance phase while the remaining 15 patients who received prednisone were tapered to ≤ 5 mg of prednisone. Among these 15 patients, nine were completely weaned off prednisone during the follow-up period while the remaining six patients were continued on ≤ 5 mg prednisone. The mean duration from stopping immunosuppression to last follow-up in this cohort was 64 months (SD, 31 months). Outcomes during follow-up are provided in Table 3. During the follow-up period, three patients experienced disease relapse involving kidney (n = 1) and lung (n = 2) at 45, 63, and 81 months post discontinuation of immunosuppressive therapy, respectively. Two of these patients were PR3-ANCA positive, and the third patient was MPO-ANCA positive. All three patients were ANCA-positive at relapse. At the time of relapse, one patient was on prednisone 3 mg daily while the remaining two patients were completely steroid-free. These three relapses were treated successfully with rituximab and prednisone. One patient had skin cancer, and another patient had thyroid cancer during the follow-up period. One patient reached end-stage renal disease and underwent a pre-emptive renal transplant. There were no deaths during the follow-up period.

**Table 3:** Clinical outcomes during follow-up IS, immunosuppression; Pos, positive; Neg, negative; Y, yes; N, no; J, joints; L, lungs; K, kidneys; N/A, not available; PID, patient identification

PID	ANCA status at time of stopping IS	Follow up time since stopping IS (months)	Relapse	Interval in months from stopping immunosuppression to relapse	ANCA status at relapse	organs involved at relapse	Malignancy after AAV diagnosis	ESRD at last follow-up	Death
1	Pos	40	N	----	----	----	N	N	N
2	Pos	61	N	----	----	----	N	1	N
3	Neg	156	N	----	----	----	N	N	N
4	N/A	81	Y	81	Pos	K	N	N	N
5	Neg	118	N	----	----	----	N	N	N
6	Pos	52	N	----	----	----	N	N	N
7	N/A	70	N	----	----	----	N	N	N
8	N/A	52	N	----	----	----	N	N	N
9	Pos	36	N	----	----	----	N	N	N
10	N/A	56	N	----	----	----	N	N	N
11	Neg	39	N	----	----	----	N	N	N
12	Neg	43	N	----	----	----	N	N	N
13	Neg	60	N	----	----	----	N	N	N
14	Neg	57	N	----	----	----	Skin	N	N
15	Pos	45	Y	45	Pos	J, L	Thyroid	N	N
16	Pos	63	Y	63	Pos	L	N	N	N
17	Neg	95	N	----	----	----	N	N	N
18	Pos	36	N	----	----	----	N	N	N

## Discussion

AAV carries a substantial risk of relapse, which is why following induction therapy with a maintenance regimen has been the standard of care since 1983 [9]. The sobering existing data on relapse risk after induction often obligates patients and their providers to opt for prolonged courses of immunosuppression. For example, the disease relapse risk was 38% at five years in a meta-analysis of European Vasculitis Study Group (EUVAS) trials using cyclophosphamide-based induction therapy [3]. Similarly, there was a 32% risk of relapse at 18 months using a rituximab-based induction therapy [10].

However, the optimal overall duration of immunosuppression and maintenance therapy specifically remain largely uncertain. The overall frequency of relapses observed in maintenance trials to date ranges from 35% to 63% [6,11-13]. Further complicating matters, long-term maintenance therapy can result in the accrual of cumulative iatrogenic toxicity over time. Such long-term adverse effects are not trivial and include cardiovascular morbidity, infection, and secondary malignancy.

Therefore, whether all AAV patients require an extended period of immunosuppression or how long patients requiring such maintenance consolidation should be treated is an issue that needs to be better defined. There is little information available to date on the outcomes of AAV patients who have had prolonged periods off immunosuppressive therapy after standard-of-care induction followed by maintenance for some defined period.

The prolonged REmis­sion-MAINtenance therapy in systemic vasculitis (REMAIN) trial is the only randomized controlled maintenance study available comparing outcomes in patients on maintenance immunosuppression versus no immunosuppression [13]. The authors concluded there appeared to be a lower relapse risk in patients who continued azathioprine, a standard maintenance agent for AAV. However, there are a number of caveats to this conclusion that should be noted. Most interestingly, two-thirds of patients in the discontinuation group did not suffer a relapse within the 48-month study follow-up period. Additionally, the number of over-treated patients in this trial was higher than usual, given the residual relapse risk in the continuation group. The data from REMAIN also demonstrated a higher incidence of infection and malignancy in the continuation group relative to the discontinuation group, a reminder of the risks of long-term therapy.

Aside from REMAIN, our single-center study is one of the few studies that have looked at outcomes in AAV patients maintained without maintenance therapy for an extended period. We acknowledge that our study is not without limitations, including the retrospective design and small sample size. In addition, the reason for stopping immunosuppression in these patients was not pre-defined and was potentially influenced by patient and physician choice. Third, our cohort was enriched with MPO-ANCA patients and those with renal involvement limiting generalization to all AAV patients. Still, despite these limitations, our study is an important representation of “real-world” practices outside a clinical trial setting. Our findings are striking as an extension of the observations about the treatment-free relapse outcomes in the REMAIN trial.

## Conclusions

AAV patients can be maintained off maintenance agents for up to three years either in the absence of prednisone therapy or with a prednisone dose not exceeding 5 mg daily. Results such as ours and from the sub-analyses from the REMAIN trail underscore a need for the AAV clinical research community as a whole to pursue in-depth studies that clearly define the need and duration of maintenance immunotherapy objectively. Ultimately, we hope such a definition of need can be incorporated into a risk-benefit formula to guide decisions about the continuation of maintenance therapy.
